# The transcriptome pattern of liver, spleen and hypothalamus provides insights into genetic and biological changes in roosters in response to castration

**DOI:** 10.3389/fgene.2022.1030886

**Published:** 2022-11-09

**Authors:** Wenpeng Li, Hui Li, Chao Yan, Siyu Chen, Xingbo Zhao

**Affiliations:** ^1^ College of Animal Science and Technology, China Agricultural University, Beijing, China; ^2^ Shenzhen Branch, Guangdong Laboratory for Lingnan Modern Agriculture, Genome Analysis Laboratory of the Ministry of Agriculture, Agricultural Genomics Institute at Shenzhen, Chinese Academy of Agricultural Sciences, Shenzhen, China; ^3^ Guangdong Provincial Key Laboratory of Animal Molecular Design and Precise Breeding, Key Laboratory of Animal Molecular Design and Precise Breeding of Guangdong Higher Education Institutes, School of Life Science and Engineering, Foshan University, Foshan, China

**Keywords:** rooster, castration, RNA-seq, metabolism, immunity

## Abstract

Chicken is widely accepted by consumers because of its delicate taste and abundant animal protein. The rooster after castration (capon) is believed to show better flavor, however, the molecular changes of the underpinned metabolism after castration is not yet understood. In this study, we aimed to figure out the alternation of meat quality and underpinned molecular mechanism *via* transcriptomic profiling of liver, spleen and hypothalamus as targeted organs in response to the castration. We identified differential expressed genes and their enriched functions and pathways in these organs between capon and rooster samples through RNA-seq analysis. In the liver, the lipid metabolism with targeted *FABP1*gene was found significantly enriched, which may be as one of the factors contributing to increased fat deposition and thus better meat flavor in capons than roosters, as predicted by the significantly lower shear force in capons than in roosters in meat quality experiments. However, the ability to xenobiotic detoxification and excretion, vitamin metabolism, and antioxidative effect of hemoglobin evidenced of the capon may be compromised by the alternation of *SULT*, *AOX1*, *CYP3A5*, *HBA1*, *HBBA*, and *HBAD*. Besides, in both the spleen and hypothalamus, *PTAFR*, *HPX*, *CTLA4*, *LAG3*, *ANPEP*, *CD24*, *ITGA2B*, *ITGB3*, *CD2*, *CD7*, and *BLB2* may play an important role in the immune system including function of platelet and T cell, development of monocyte/macrophage and B cell in capons as compared to roosters. In conclusion, our study sheds lights into the possible molecular mechanism of better meat flavor, fatty deposit, oxidative detoxification and immune response difference between capons and roosters.

## Introduction

As known, China has become the second largest chicken producer in the world. In China, chicken meat source includes meat from fast-growing, medium-growing, and slow-growing chicken referring to native breeds for dual-purpose of both meat and eggs. Except that, the capon meat is also popular, especially in the South China. Castration, referring to the removal or any intervention that causes loss of function of testicles or ovaries, usually results in the loss of the reproductive function. The capons, tracing back to more than 2,000 years ago (Franco Ruiz et al., 2016), originated in southern China, and then spread to different countries such as Italy, Poland, France, Japan, the United States, etc. (Sirri et al., 2009; Calik, 2014; Sokołowicz et al., 2015). Due to technical limitation, capons can only be applied for a small scale, nevertheless, some of the stockperson and consumers agree that capons have better meat characteristics and they are more likely to buy capon meat (Franco Ruiz et al., 2016). The removal of the testicles, usually leads to changes in the secondary sexual characteristics, such as the reduction of the size of the crown and wattles. The most important physiological effect caused by castration is the decrease of plasma testosterone levels (Symeon et al., 2010; Lin et al., 2012). The body shape of the capons is more graceful, and the neck feathers, saddle feathers, and tail feathers are changed to longer and more vivid. Afterward, the growth and carcass performance are improved, and the meat becomes more tender and juicier. Besides, capons usually shows less fighting behavior (Chen et al., 2006), which is also associated to more feed reward and deposit fat. Further, the meat flavor is improved by the increase of intermuscular and subcutaneous fat (Riha and Ho, 1998), which may be one of the factors contributing to the meat quality of capons (Tor et al., 2005). The changes of meat quality and flavor after the castration have already been reported, but its underpinned molecular mechanism remains unknown.

Capons have higher cholesterol and triglyceride concentrations, which is related to the increased liver fat production. A previous study has analyzed the gene expression profiles of intact rooster liver, and indicates that androgen status has a negative regulatory effect on phosphoenolpyruvate carboxykinase 1 (*PCK1*) gene, which is involved in liver fat biosynthesis (Duan et al., 2013). Although the molecular mechanisms of fat deposition affected by testosterone level have remained incompletely understood, caponization affecting lipid metabolism has been confirmed (Symeon et al., 2013; Mašek et al., 2014; Cui et al., 2018). Testosterone deficiency induced by castration affect the innate and adaptive immune systems (Ellis et al., 2001; Heng et al., 2005; Sutherland et al., 2005; Rettew et al., 2008; Meier et al., 2009). Androgens are generally immunosuppressive, testosterone deficiency induced by castration acting on many aspects of the immune system (Trigunaite et al., 2015). Besides, the hypothalamus is an evolutionarily ancient collection of deep subcortical nuclei that control homeostatic, innate “survival behaviors” and also associated with immune and sympathetic nervous system (Saper and Lowell, 2014). The drop in testosterone levels caused by castration can also lead to a series of downstream reactions by negative feedback regulation, including insulin resistance, impaired glucose tolerance, fatty liver formation, reduced energy expenditure, leptin resistance, etc. (Morford et al., 2018).

That is therefore, this study was first to investigate the effect of castration on meat quality, and aimed to focus on the liver, spleen and hypothalamus as targeted organs and find out the underpinned molecular mechanism *via* transcriptomic profiling in response to castration. Our study will help in the understanding of the changes of molecular mechanism of roosters that induced by castration and provide a responsible manner to consume capon meat.

## Materials and methods

### Ethics and statement

The experimental and animal care protocols were approved by the China Agricultural University Laboratory Animal Welfare and Animal Experimental Ethical Inspection (approval number: CAU20210525-1).

### Experimental animals and sample collection

Forty chickens of Weining chicken breed, a Chinese dual-purpose native breed, were randomly collected from the same barn and reared under the same cage system during the brooder period. The chicks were castrated at 20-day-old following a commercial poultry industry protocol, and thus divided into rooster group (*n* = 20) and capon group (*n* = 20). Feed and water were deprived of 12 h before the surgery. In briefly, feathers are removed around the incision site located between the last two ribs, and then the skin is sterilized. A 1.5 cm incision is made and the testis is removed with a rib retractor. The incision site is then disinfected and sutured. The effectiveness of the castration is checked during slaughter during the evisceration procedure. For those experimental animals, they were reared together under the free-ranging in 20-square-meter enclosures at a density of two animals per square meter. Basal diets were commercial concentrates with *ad libitum* feed and water.

At 120 days of the age, three capons and three roosters that were randomly selected from the population and killed at a local commercial broiler slaughterhouse. The liver, spleen and hypothalamus samples were collected immediately after the slaughter. After liquid nitrogen freezing, the tissue samples were stored at −80°C for further transcriptome (RNA-Seq) and qRT-PCR analyses. The transcriptome and qRT-PCR analysis were set up three biological replicates. The remaining samples were stored at −80°C for standby. Hypothalamus, liver, spleen of roosters was abbreviated as H_R, L_R, S_R, while hypothalamus, liver, spleen of capon was namely for H_C, L_C, S_C, respectively.

### Meat quality analysis

#### pH

Measurements of pH values were carried out using a portable pH probe (PH-STAR, Matthaus, Germany) by inserting the probe into the center of the muscle sample within 24 h after slaughter. The pH meter was calibrated prior to the measurement using two buffer solutions of pH 4.0 and 7.0. The probe was inserted into the muscle three times (in different locations) and the final pH value used was the mean of the three measurements.

#### Drip Loss

To determine drip loss, meat samples were weighed before analysis. Each sample was then suspended in a sealed polyethylene bag with an aluminum wire hook and sewing thread. The sealed bag was filled with nitrogen to minimize the contact between the meat sample and the bag. Both were then suspended in a refrigerator at 4°C. After storage for 24 h, the surface water of the meat was gently dried with filter paper before weighing. The drip loss values were calculated using the difference between the initial and final weights of the samples, with the results expressed as a percentage of the initial weight (Honikel, 1997).

#### Cooking loss

To determine cooking loss, samples were pre-weighed and subsequently placed in sealed polyethylene bags. The bags were then transferred into continuously heated boiling water until the samples reached an internal temperature of 75°C, as measured by a portable thermometer (Hengko, Shenzhen, China). The bags were then removed from the boiling water and kept at 25°C. The portable thermometer was used to determine when the meat sample had cooled to 25°C, at which point surface water was gently wiped from the sample using a paper towel. Cooking loss values were calculated as the difference between the initial and final weights of the samples, with the results expressed as a percentage of the initial weight (Pinheiro et al., 2019).

#### Shear Force

To measure shear force, the same samples from the cooking loss measurements were used. They were placed overnight at 4°C in a fridge (BCD-251U, Hisense, China) and then removed and cut into 1 × 1 × 4 cm long strips to determine the shear force using a muscle tenderness meter (C-LM3B, Tenovo, China) by Warner-Bratzer method (Pinheiro et al., 2019). The values collected were a mean of five measurements and are expressed in kgf.

### RNA extraction and quality detection

Total RNA was extracted from the tissue using TRIzol^®^ Reagent according the manufacturer’s instructions (Invitrogen) and genomic DNA was removed using DNase I (TaKara). Then RNA quality was determined by 2100 Bioanalyser (Agilent) and quantified using the ND-2000 (NanoDrop Technologies). Only high-quality RNA sample (OD260/280 = 1.8–2.2, OD260/230 ≥ 2.0, RIN ≥6.5, 28S:18S ≥ 1.0, > 2 μg) was used to construct sequencing library.

### RNA-seq library construction and sequencing

RNA-seq transcriptome library was prepared following TruSeq™ RNA sample preparation Kit from Illumina (San Diego, CA) using 1 μg total RNA. Shortly, messenger RNA was isolated according to polyA selection method by oligo (dT) beads and then fragmented by fragmentation buffer firstly. Secondly double-stranded cDNA was synthesized using a SuperScript double-stranded cDNA synthesis kit (Invitrogen, CA) with random hexamer primers (Illumina). Then the synthesized cDNA was subjected to end-repair, phosphorylation and “A” base addition according to Illumina’s library construction protocol. Libraries were size selected for cDNA target fragments of 200–300 bp on 2% Low Range Ultra Agarose followed by PCR amplified using Phusion DNA polymerase (NEB) for 15 PCR cycles. After quantified by TBS380, paired-end RNA-seq sequencing library was sequenced with the Illumina HiSeq xten/NovaSeq 6000 sequencer (2 × 150 bp read length).

### Analysis of differentially expressed genes

Based on the FPKM values of the Illumina sequencing data, mRNA expression levels in six different libraries of the two groups were evaluated, and differential expression analysis was performed using the DESeq2 R v1.14.1package. The *p* value was adjusted using Benjamini and Hochberg to control FDR. In this study, FPKM >1, adjusted *p*-value (padj) < 0.05 and |log2fold changes| ≥ 1 were defined as significant differential expression genes (DEGs). The heat map clustering analysis of DEGs was performed using a pheatmap R package.

### Gene ontology and KEGG enrichment analysis

The bioinformatics analysis methods were used to perform Gene Ontology (GO) enrichment analysis and Kyoto Encyclopedia of Genes and Genomes (KEGG) pathway analysis on DEGs. Using the software Goatools (v0.6.5) to perform GO enrichment analysis in the gene set to obtain the main GO functions of the genes. The statical method is Fisher’s exact test. When the corrected *p*-value (q value) < 0.05, the GO function is considered to be significantly enriched. KEGG pathway annotation is the main public pathway database for understanding the biological functions of genes, and the database permits the systematic analysis of gene function and genome information. The R package was used to perform KEGG pathway enrichment analysis of DEGs. The calculation principle is the same as the GO function enrichment analysis. When the corrected *p*-value (q value) < 0.05, the KEGG pathway function is considered to be significantly enriched.

### qRT-PCR analysis of DEGs

We randomly selected 12 differentially expressed genes from the three tissues, including six up- and down regulated genes, to verify the accuracy of the sequencing data using qRT-PCR. Total RNA from each sample was extracted with EASYspin Plus kit (Aidlab, Beijing, China) and then reverse transcribed into cDNA with TRUEscript RT MasterMix kit (Aidlab, Beijing, China) according to the manufacturer’s instructions. The specific primers for DEGs were designed online using the Primer-BLAST program of NCBI, and the chicken *GAPDH* gene was used as the internal reference gene. Quantitative reverse transcription polymerase chain reaction was performed using Bio-Rad CFX-96 (Bio-Rad, United States). The reaction system was 25 μL, including 12.5 μL of 2 x SYBR Green qPCR Mix (Aidlab, Beijing, China), 10.5 μL of RNase-free water, 0.5 μL of forward and reverse primers, and 1 μL of cDNA. The qRT-PCR program was as follows: 95°C 2 min; 95°C 15 s; 60°C 15 s and 72°C 30 s, a total of 40 cycles. A standard curve is plotted based on the gradient sample concentration and the corresponding Cq value. The amplification efficiency was determined by the slope of the standard curve (k) using $E=10^{\left(-\frac{1}{k}\right)}-1$ . The relative expression of mRNA was calculated by 2^−ΔΔCT^ method. The qRT-PCR samples were identical to the RNA-seq samples, with 3 biological replicates per group and 3 technical replicates per sample. Primer sequences and amplification efficiency are shown in Supplementary Table S1.

### Statistical analysis

The experimental data were presented as the mean ± standard error of three repetitions. GraphPad Prism (version 5.0) software (San Diego, CA, United States) was used for statistical analysis of the qRT-PCR graphs. Data of meat quality did not meet the assumptions for parametric analysis, and no variable could be successfully transformed to meet these assumptions. Thus, the difference of these data between the two experimental groups was analyzed using non-parametric tests of two independent samples (Mann-Whitney U test) with *p* < 0.05 classified as indicating a significant difference. The analyses were performed using SPSS 20.0 software (IBM, United States).

## Results

### Physical properties of the meat

The effect of caponization on meat quality attributes is presented in Table 1. The pH value, drip loss, cook loss were not affected by the castration, however, the castration increased shear force value (*p* < 0.05).

**TABLE 1:** Difference of meat quality between capons and roosters.

	Rooster	Capon	*p*-value
pH	6.15 ± 0.28	6.06 ± 0.23	0.69
Drip Loss (%)	2.24 ± 0.80	3.00 ± 0.71	0.31
Cooking Loss (%)	32.49 ± 3.40	36.93 ± 2.11	0.10
Shear Force (kgf)	34.51 ± 3.96	26.95 ± 3.24	0.01

### Summary of transcriptome sequencing data quality

Illumina HiSeq sequencing results showed that 18 libraries from the two groups generated raw reads and retained clean reads by removing the low-quality reads are as below. The comparison ratio of the transcriptome and the reference genome (*Gallus gallus*) in each sample is greater than 75%, and the multiple mapped is less than 10%, indicating that the reference genome is selected properly. The experimental sample is free of contamination, the data is valid, and the differential expression of the transcriptome identification gene has high reliability and accuracy (Supplementary Table S2).

### RNA-seq results, DEGs identification and functional annotation

#### Analysis of differentially expressed genes in liver

A total of 102 DEGs (*p*adj < 0.05, |log2fold changes| ≥ 1 and FPKM >1) were found between the capons and roosters. Among them, 47 genes were upregulated and 55 genes were downregulated. The top 10 most significantly upregulated and downregulated genes were shown in Table 2. Among the differentially expressed genes, the gene with the highest up-regulated expression fold is fatty acid binding protein 1 (*FABP1*), and the gene with the highest down-regulated expression fold sulfotransferase (*SULT*). The cluster analysis results of DEGs suggested that the gene express patterns were similar within the group but different between the groups (Supplementary Figure S1).

**TABLE 2:** The top 10 most significantly up- and down-regulated genes in the liver between the capon and rooster group.

Upregulated	Log2FC	*P*adj	Downregulated	Log2FC	*P*adj
*FABP1*	3.163	<0.001	*SULT*	−3.662	<0.001
*C12orf75*	2.885	<0.001	*WBP1L*	−1.039	<0.001
*A2ML1*	2.282	<0.001	*CHIA-M31*	−2.938	<0.001
*SEBOX*	2.630	<0.001	*EPAS1*	−1.436	<0.001
*MSMB*	3.066	<0.001	*ANK1*	−1.671	<0.001
*CSRP1*	1.281	0.002	*AOAH*	−1.042	<0.001
*ASMTL*	1.124	0.004	*PTPN1*	−1.053	0.001
*SPTBN5*	3.519	0.006	*TIMP3*	−1.758	0.001
*DCDC2*	2.229	0.007	*ADM*	−1.547	0.002
*DBI*	1.026	0.007	*SCN4A*	−1.416	0.003

#### Gene ontology analysis of DEGs in liver

**FIGURE 1:**
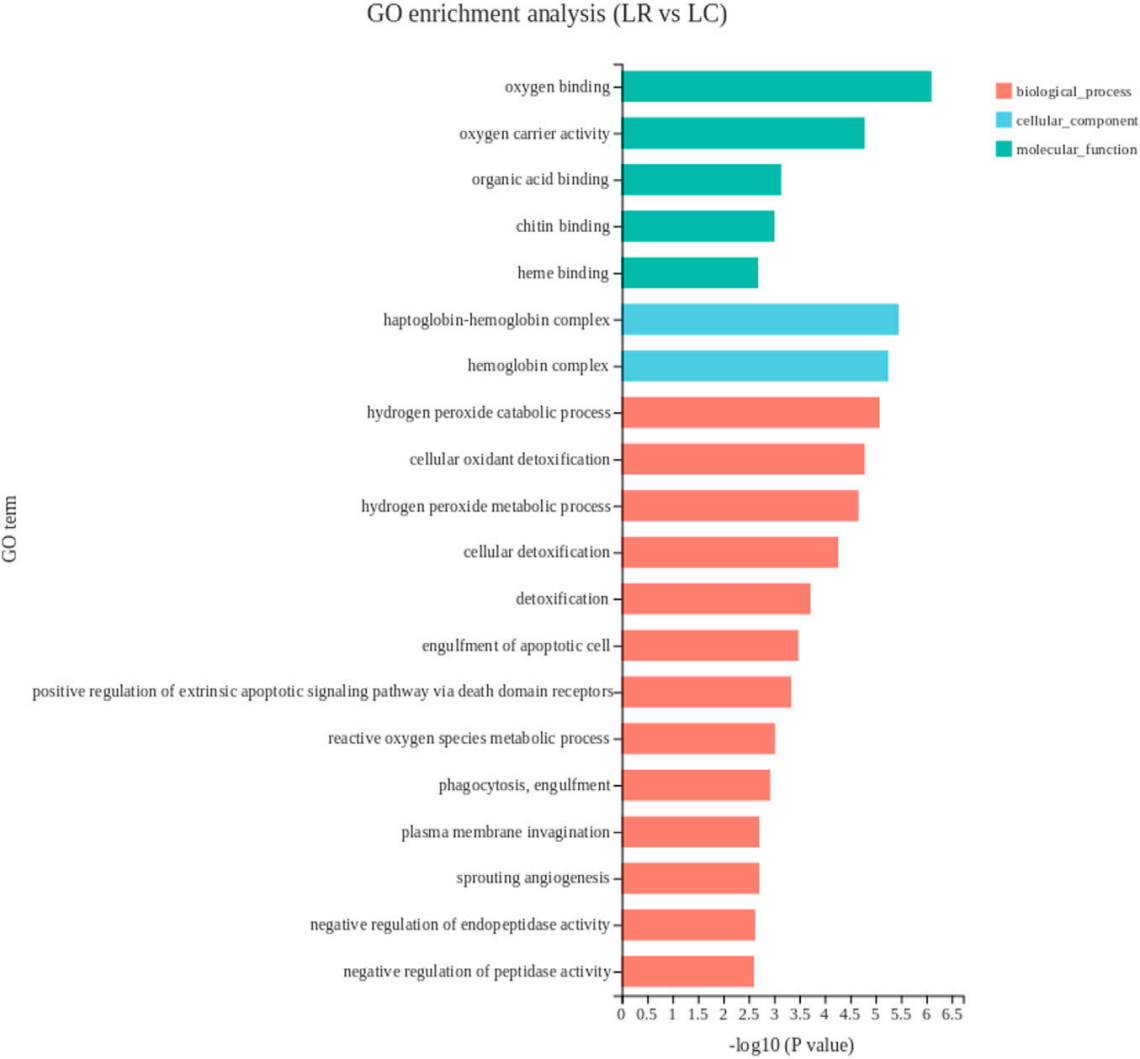
The top 20 enriched gene ontology terms of DEGs in liver. The *X*-axis shows the significance level of the enrichment, corresponding to the height of the column, where the smaller the FDR and the greater the −log10 (*p*-value) value, the more significantly enriched the GO term; the *Y*-axis shows the DEG-enriched GO term. LR, liver of rooster; LC, liver of capon.

To further analyze the function of DEGs, GO enrichment analysis was performed. The result showed that the DEGs in the liver tissue were enriched on 231 GO terms. The top 20 GO terms were shown in Figure 1. Worthy notably, in the biological process, the primary subcategories were the hydrogen peroxide catabolic process, cellular oxidant detoxification and hydrogen peroxide metabolic process. The metabolic system was the most affected within the subcategories.

#### KEGG pathway enrichment analysis of DEGs in liver

To identify genes involved in the regulation of metabolism and their pathways, we performed KEGG pathway analysis on DEGs.

**FIGURE 2:**
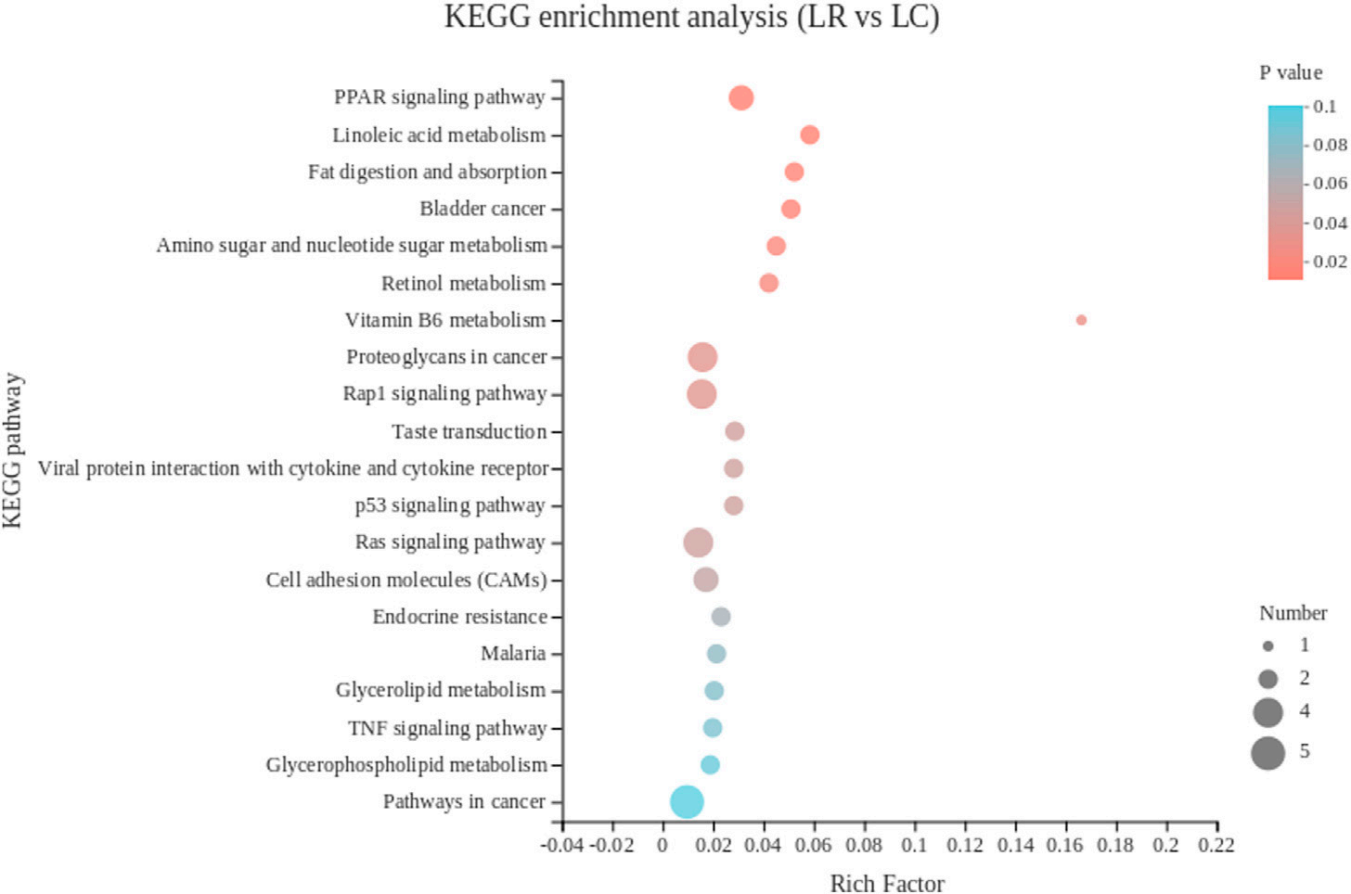
The top 20 Kyoto Encyclopedia of Genes and Genomes pathways of DEGs in liver. The color of the dot represents the *p* value, and the size of the dot represents the number of DEGs enriched in the reference pathway. LR, liver of rooster; LC, liver of capon.

The 102 DEGs were enriched in 104 KEGG pathways, and the top 20 KEGG pathways were showed in Figure 2. The lipid-metabolism-related pathways include PPAR signaling pathway, fat digestion and absorption, and linoleic acid metabolism. The vitamin-metabolism-related pathways include retinol metabolism and vitamin B6 metabolism. Among them, DEGs including *DBI* and *FABP1* were enriched in the PPAR signaling pathway, *AOX1* and *CYP3A5* were enriched in retinol metabolism, *FABP1, PLA2G3* were enriched in fat digestion and absorption, *AOX1* was enriched in vitamin B6 metabolism, and *CYP3A5* and *PLA2G3* were enriched in linoleic acid metabolism.

#### Analysis of differentially expressed genes in spleen

A total of 780 DEGs (*p*adj < 0.05, |log2fold changes| ≥ 1 and FPKM >1) were selected between the capon group and rooster group, of which 376 genes were upregulated and 404 genes were downregulated. The top 10 genes that significantly upregulated and downregulated were shown in Table 3. Among the 780 DGEs, the gene with the highest up-regulated expression fold is Ermin (*ERMN*). The gene with the highest down-regulated expression fold is platelet activating factor receptor (*PTAFR*). Supplementary Figure S2 showd the cluster analysis results of DEGs, which suggested that the gene express patterns were similar within the group but different between the groups.

**TABLE 3:** The top 10 significantly up- and down-regulated genes in the spleen of the capon group compared to that of rooster group.

Upregulated	Log2FC	*P*adj	Downregulated	Log2FC	*P*adj
*ERMN*	10.302	<0.001	*ST8SIA6*	−3.48	<0.001
*WDR6*	2.111	<0.001	*EFCC1*	−3.390	<0.001
*SOD3*	1.697	<0.001	*SMPD3*	−2.283	<0.001
*POLD4*	4.905	<0.001	*CAT*	−1.632	<0.001
*HPX*	2.505	<0.001	*AKAP12*	−1.924	<0.001
*APOA1*	1.579	<0.001	*FSHR*	−1.810	<0.001
*IGFBP5*	1.876	<0.001	*PTAFR*	−10.503	<0.001
*CHGB*	1.593	<0.001	*DOCK5*	−2.123	<0.001
*CSPG4*	1.705	<0.001	*PIEZ O 2*	−2.325	<0.001
*RAB7B*	1.991	<0.001	*ANK3*	−2.326	<0.001

#### Gene ontology analysis of DEGs in spleen

**FIGURE 3:**
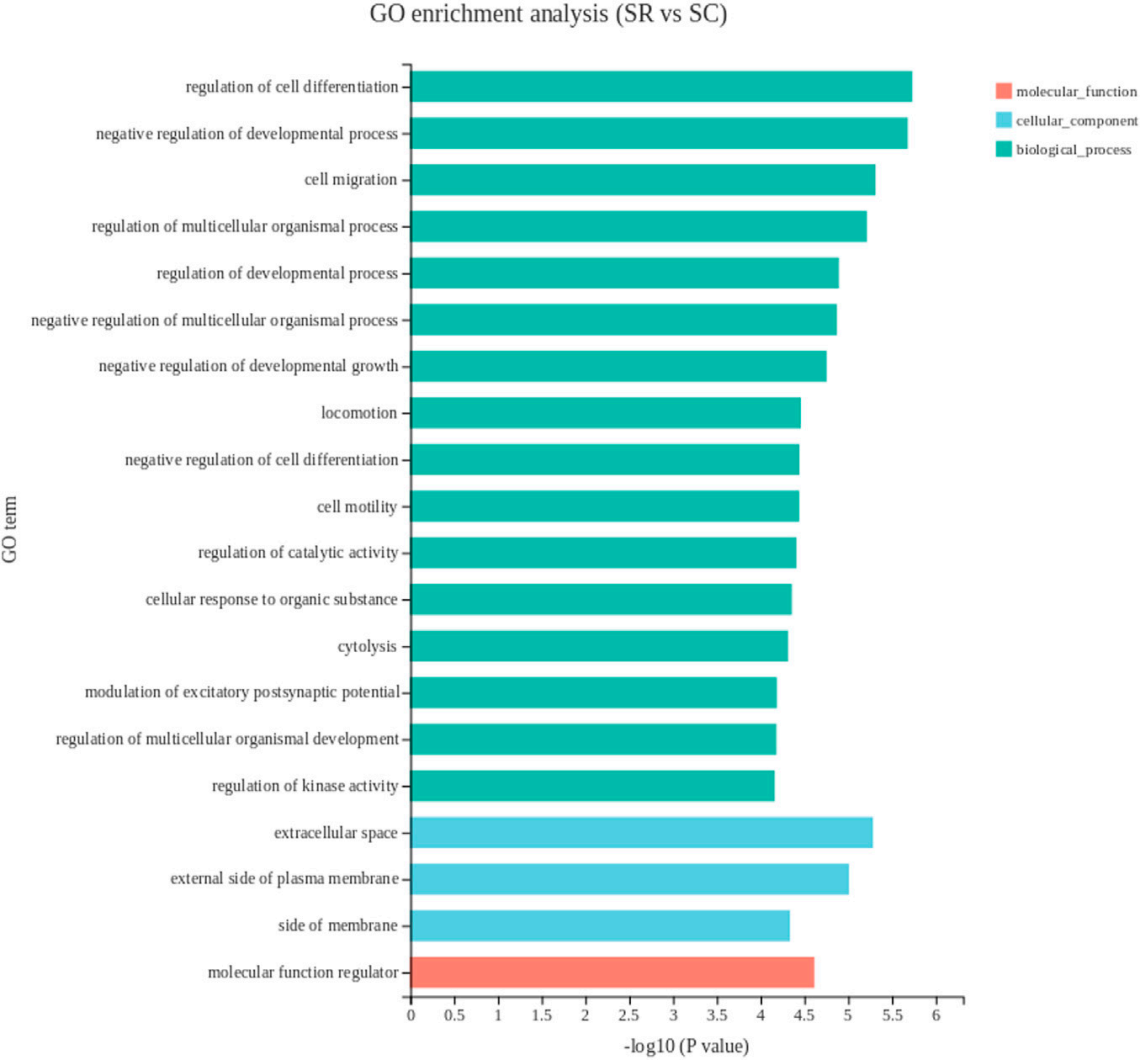
The top 20 enriched gene ontology terms of DEGs in spleen. The *X*-axis shows the significance level of the enrichment, corresponding to the height of the column, where the smaller the FDR and the greater the −log10 (*p*-value) value, the more significantly enriched the GO term; the *Y*-axis shows the DEG-enriched GO term. SR, spleen of rooster; SC, spleen of capon.

The DEGs in the spleen were enriched for 740 GO terms. The top 20 GO terms were shown in Figure 3. Most DEGs were involved in biological process. The primary subcategories were the regulation of cell differentiation, which contains upregulated genes *APOA1, PITHD1, ID1, THY1, CTLA4, LAG3* etc*.*, downregulated genes *GPR171*, *TIAM2*, *TIAM1*, *CRTAM* etc.

#### KEGG pathway enrichment analysis of DEGs in spleen

**FIGURE 4:**
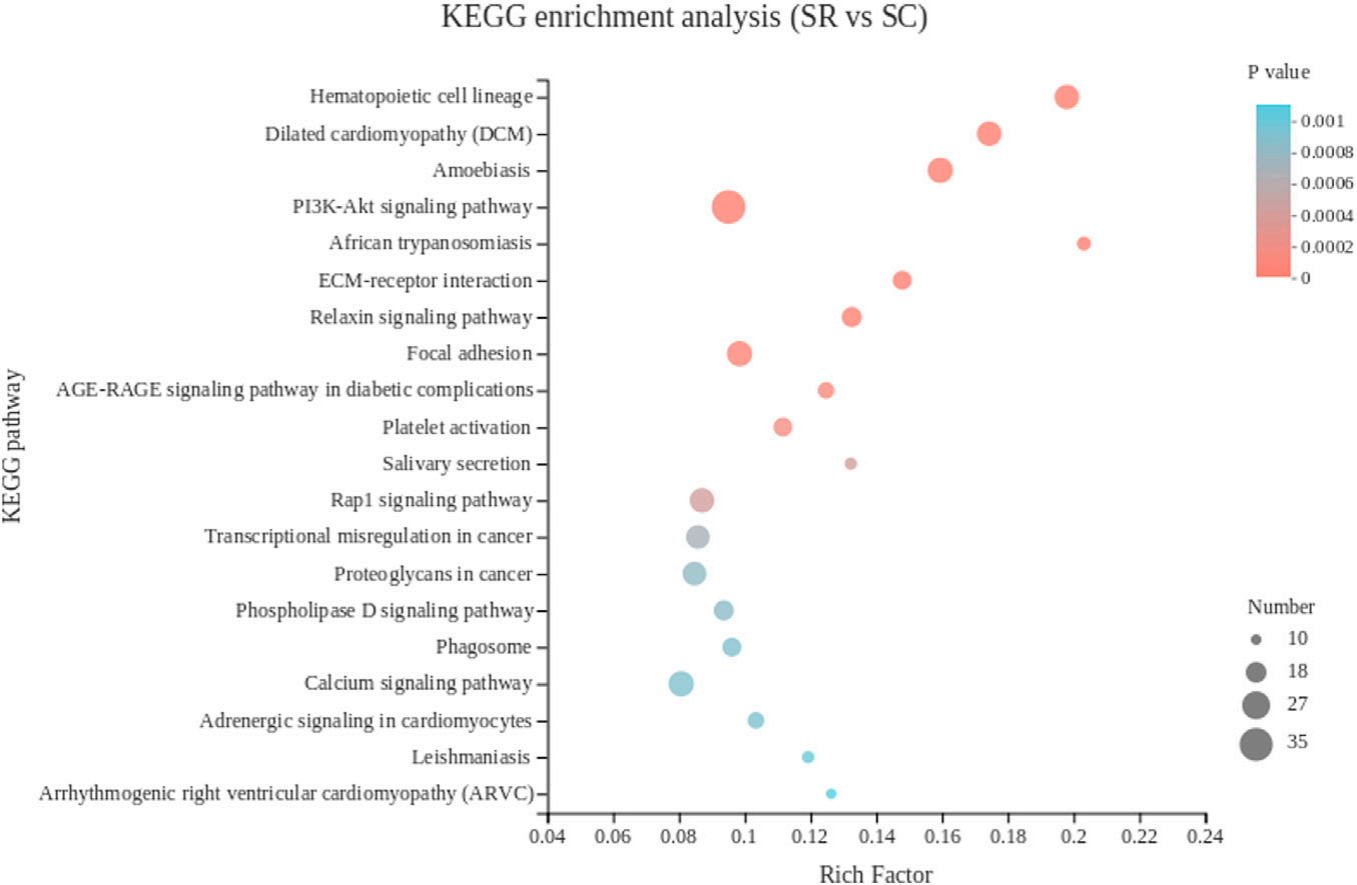
The top 20 Kyoto Encyclopedia of Genes and Genomes pathways of DEGs in spleen. The color of the dot represents the *p* value, and the size of the dot represents the number of DEGs enriched in the reference pathway. SR, spleen of rooster; SC, spleen of capon.

The KEGG pathway analysis showed that 780 DEGs of spleen tissue were enriched in 274 pathways, and the top 30 pathways were showed in Figure 4. The most significantly enriched pathway is hematopoietic cell lineage, the immune-related genes *CD1c*, *ANPEP*, *CD2*, *CD7*, *CD24*, *IL5RA*, *ITGA6*, *ITGB3* and other 13 DEGs were enriched.

#### Analysis of differentially expressed genes in hypothalamus

There are only 15 DEGs (*p*adj < 0.05, |log2fold changes| ≥ 1 and FPKM >1) between the capon group and rooster group. Of which 11 DEGs were upregulated and four DEGs were downregulated (Table 4). Except for the uncharacterized gene, upregulated genes contain leukocyte ribonuclease A-2 (*RSFR*), RNA, 7SL, cytoplasmic 1(*RN7SL1*) and major histocompatibility complex class II beta chain BLB2 (*BLB2*); downregulated genes contain kainate binding protein (*KBP*), collagen type XXVIII alpha 1 chain, transcript variant X2 (*COL28A1*) and ETS transcription factor, transcript variant X1 (*FEV*). The cluster analysis results of DEGs suggested that the gene express patterns were similar within the group but different between the groups (Supplementary Figure S3).

**TABLE 4:** The top 10 genes in the hypothalamus of the capon group were up- and down-regulated compared to that of rooster group.

Upregulated	Log2FC	*P*adj	Downregulated	Log2FC	*P*adj
*RN7SL1*	1.771	<0.001	*LOC101749214*	4.527	<0.001
*LOC121107020*	1.239	<0.001	*COL28A1*	−1.252	0.006
*LOC121111962*	1.860	<0.001	*FEV*	−4.111	0.015
*RSFR*	2.249	<0.001	*KBP*	−1.121	0.017
*MIR1784B*	3.610	0.001			
*LOC112530295*	2.421	0.004			
*LOC121112595*	6.838	0.005			
*LOC121111963*	1.361	0.014			
*LOC121112259*	1.137	0.014			
*BLB2*	1.208	0.026			

#### Gene ontology analysis of DEGs in hypothalamus

**FIGURE 5:**
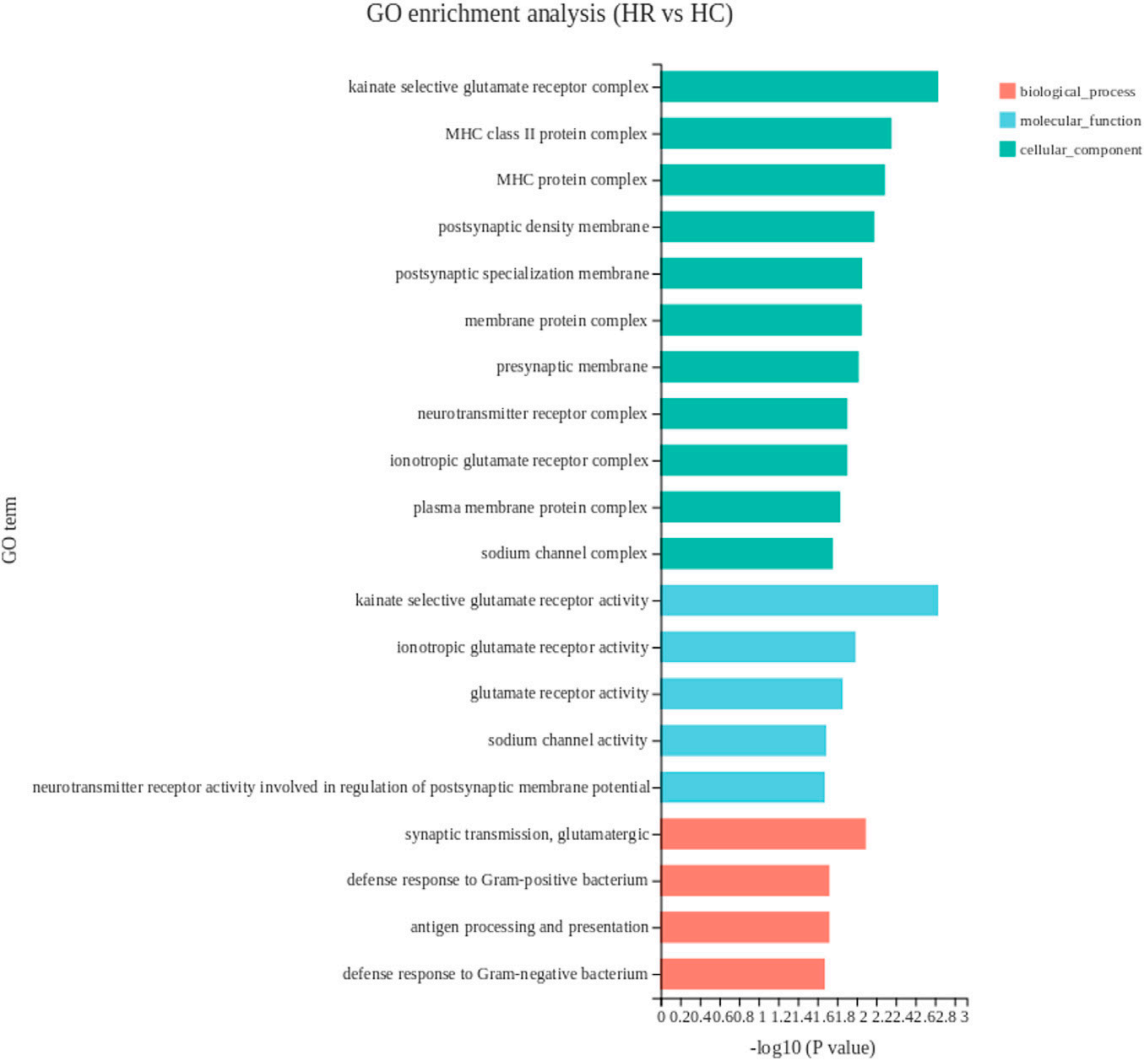
The top 20 enriched gene ontology terms of DEGs in hypothalamus. The *X*-axis shows the significance level of the enrichment, corresponding to the height of the column, where the smaller the FDR and the greater the -log10 (*p*-value) value, the more significantly enriched the GO term; the *Y*-axis shows the SDEG-enriched GO term. HR, hypothalamus of rooster; HC, hypothalamus of capon.

Between the hypothalamus tissue of capon and rooster groups, 15 DEGs were enriched on 38 GO terms, the top 20 GO terms were listed in Figure 5. DEGs in the hypothalamus between the two experimental groups were mainly enriched in the cellular component including MHC class II protein complex, MHC protein complex and kainate selective glutamate receptor complex. Among all the significantly enriched GO terms, and *BLB2, RN7SL1, RSFR* were upregulated while genes *KBP, COL28A1* were downregulated.

#### KEGG pathway enrichment analysis of DEGs in hypothalamus

**FIGURE 6:**
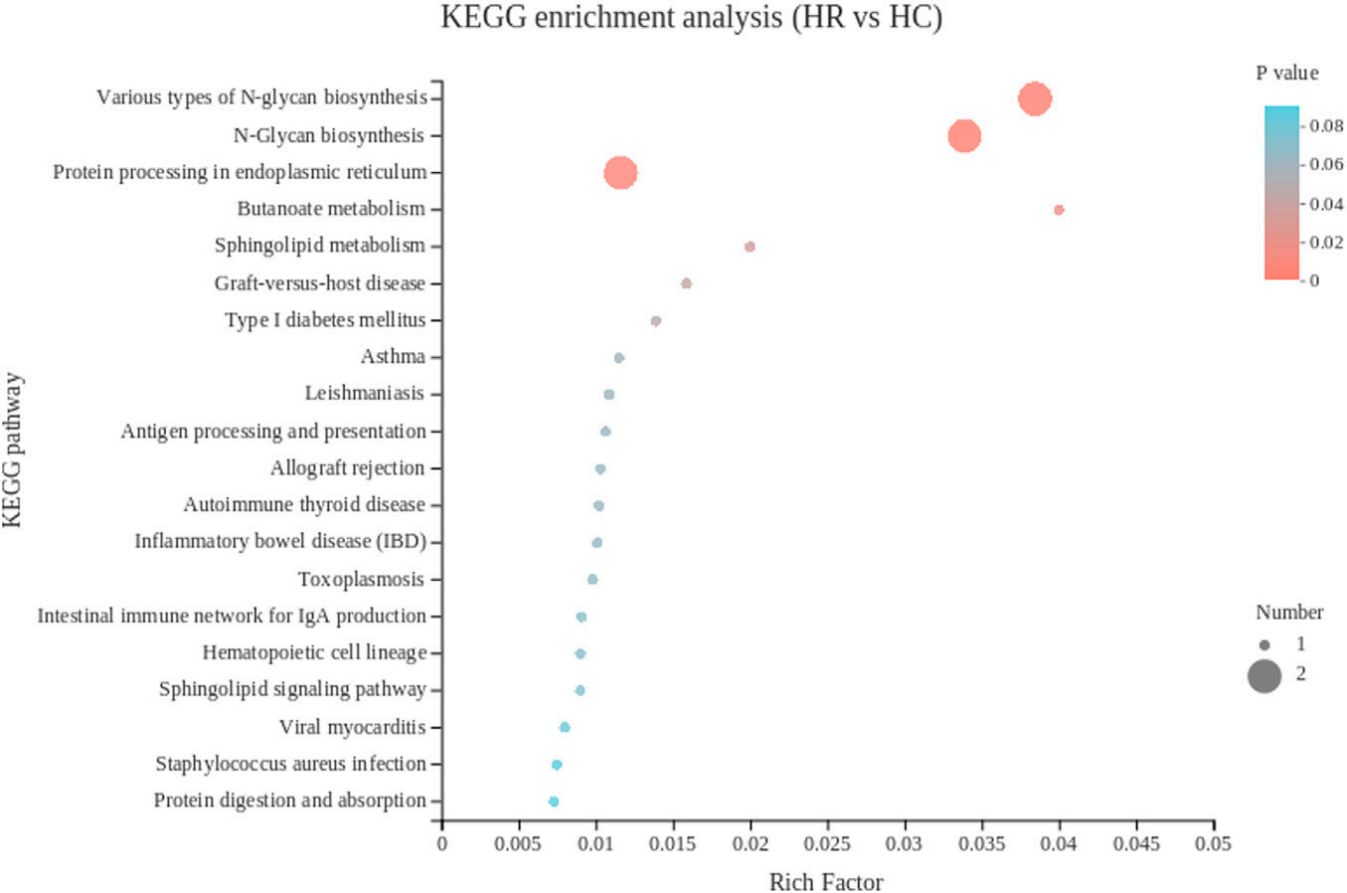
The top 20 Kyoto Encyclopedia of Genes and Genomes pathways of DEGs in hypothalamus. The color of the dot represents the *p* value, and the size of the dot represents the number of SDEGs enriched in the reference pathway. HR, hypothalamus of rooster; HC, hypothalamus of capon.

The KEGG pathway analysis of hypothalamus showed that 15 DEGs were enriched in 32 pathways, and the top 30 KEGG pathways were showed in Figure 6. The most significant enrichment pathways included Various types of N-glycan biosynthesis and N-Glycan biosynthesis. *BLB2* was significantly enriched in pathways including graft-versus-host disease and Type I diabetes mellitus. Metabolism-related gene *RN7SL1* was significantly enriched in the butanoate metabolism and sphingolipid metabolism.

### Verification of DEGs by qRT-PCR

**FIGURE 7:**
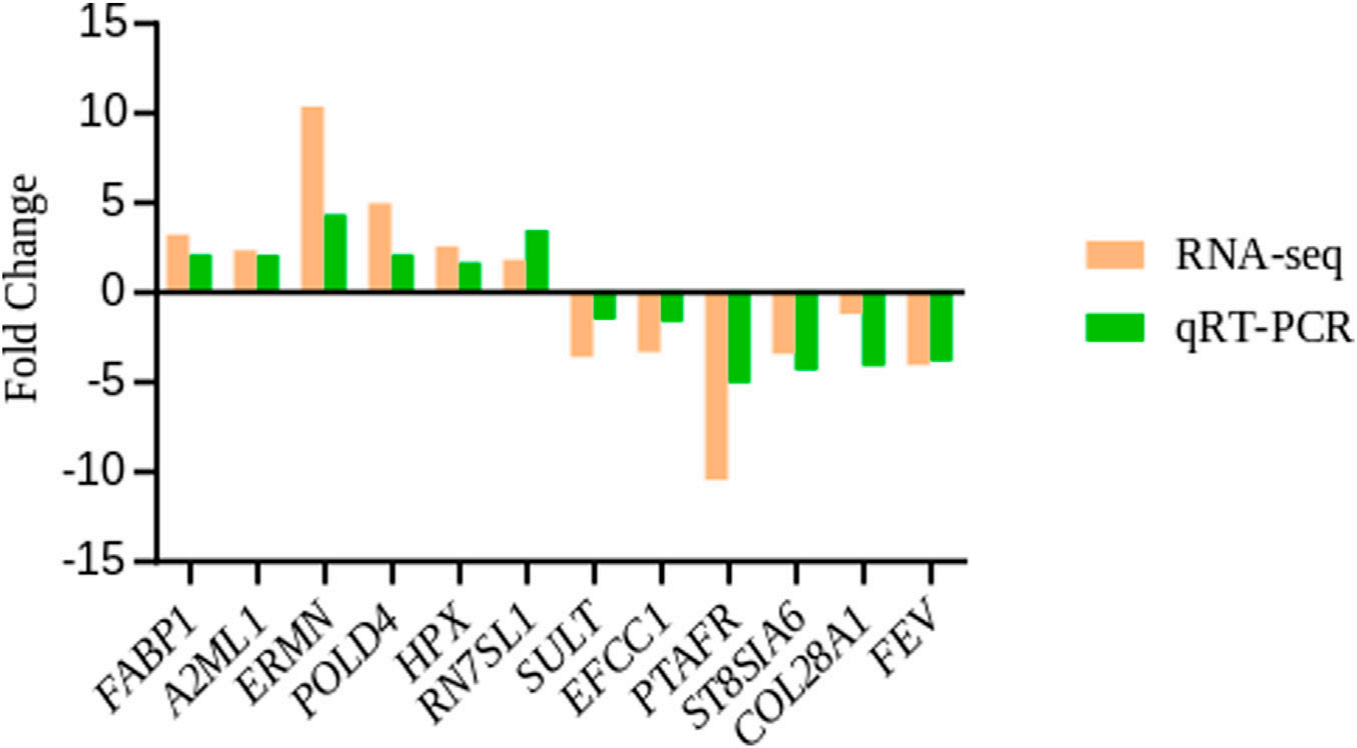
qRT-PCR validation of differentially expressed genes from liver, spleen and hypothalamus between capons and roosters. *β-actin* was used as an internal control, data were presented as fold change (*n* = 3 per group).

Twelve candidate genes were screened for qRT-PCR analysis. These genes included two up-regulated (*FABP1* and *A2ML1*) and one down-regulated in liver (*SULT*); three up-regulated (*ERMN*, *POLD4* and *HPX*) and three down-regulated genes (*ST8SIA6*, *EFCC1* and *PTAFR*) in the spleen; and one gene up-regulated (*RN7SL1*) and two down-regulated genes in the hypothalamus (*COL28A1* and *FEV*). The observed expression trends confirmed the RNA-seq results (Figure 7), indicating that the RNA-seq results were reliable.

## Discussion

### Physical properties of the meat

The results obtained in the current study were in line with other study showing no significant effects of caponization on pH value or water-holding capacity (Cui et al., 2016). pH values were mainly affected by anaerobic glycolysis during slaughter (Liu et al., 2020) and muscle water-holding capacity was affected by the degeneration of myofibril protein and sarcoplasmic protein (Hughes et al., 2014). Therefore, the result suggested that castration have no effect on pH and water-holding capacity. Besides, the present study shows that the shear force value was lower in the capon group than that in the rooster group, which was consistent with a previous investigation (Cui et al., 2016). It is widely accepted that caponization lead to fat deposition and androgens inhibit fat deposition (Duan et al., 2013), different shear force value may be due to by different fat content in two groups (Amorim et al., 2016).

### Differences in liver transcriptomic profiles between capons and roosters

In this study, a total of 102 DEGs were detected in liver. As the most significantly up-regulated gene, *FABP1* also known as *L-FABP*, its ablation greatly enhances many effects of dietary cholesterol, thereby inducing the accumulation of liver cholesterol (mainly cholesterol ester) and triacylglycerol, and increasing the weight gain in mice and chicken (Martin et al., 2006; Wang et al., 2006; Park and Sung, 2015). Furthermore, other studies have shown that overexpression of *FABP1* significantly increases the uptake of fatty acids by hepatocytes (Wu et al., 2016), and the inhibition of *FABP1* reduces fatty acid uptake, key rate-limiting enzymes in fatty acid biosynthesis, and increases fatty acid β-oxidation (Mukai et al., 2017). In this study, *FABP1* was upregulated in the liver of capons, which we speculated that this is affected by the alternation of fat deposition after caponization. As the most downregulated gene in liver, sulfotransferase (*SULT*) plays an important role in the xenobiotic detoxification of chicken livers (Ahn et al., 2019).

In this study, most of the DEGs in liver were enriched in biological process in the GO analysis. In biological process category, the top three pathways were all associated with oxidative detoxification. *HBA1*, *HBBA*, *HBAD*, were significantly enriched and mapped to hydrogen peroxide catabolic process, cellular oxidant detoxification and hydrogen peroxide metabolic process, which were all related to the recovery of oxidative damage caused by reactive oxygen species (ROS). All three genes encode different subunits of hemoglobin, which reduces DNA repair by reducing DNA double-strand breaks (Gafter-Gvili et al., 2013). In addition, plasma total antioxidant capacity was positively correlated with hemoglobin levels (Campise et al., 2003; Aslan et al., 2006), Both plasma total antioxidant capacity and hemoglobin level were negatively correlated with lymphocyte DNA damage (Aslan et al., 2006). The down-regulated expression of these genes after castration may indicate that capon has a lower capacity of antioxidant defense. This is in agreement with a previous research reported that testosterone contributed to upregulate the bioavailability of certain antioxidants (Noguera et al., 2011). In KEGG pathway analysis, the first three pathways with the most significant enrichment were all related to lipid metabolism, genes enriched in these pathways with differences in expression include *FABP1*, *PLA2G3* and *CYP3A5*.*FABP1* was enriched in all lipid metabolism related pathways, according to the function of *FABP1* has been introduced above, which suggests *FABP1* has a great effect on lipid metabolism in the liver of capon. It has been reported that *PLA2G3* hydrolyzes all phospholipid subclasses linking to phosphatidylcholine, phosphatidylethanolamine, phosphatidylserine, phosphatidylinositol and phosphatidylglycerol (Murase et al., 2017). Besides, cholesterol is metabolized to 4β-hydroxycholesterol or 25-hydroxycholesterol by *CYP3A5* (Nylén et al., 2011; Nitta et al., 2018). Also, steatosis is associated with a significant reduction in hepatic *CYP3A* activity *in vitro* (Kolwankar et al., 2007). Therefore, we assumed that the meat flavor of capon is better than rooster because numerous studies have shown that fatty acids contribute to the flavor of meat (Sun et al., 2022). Among the top 20 pathways, the enrichment of DEGs in retinol metabolism and vitamin B6 metabolism pathways are second only to fat metabolism-related pathway. *AOX1* encodes an aldehyde oxidase that oxidizes pyridoxal (a form of vitamin B6) to pyridoxine, which is then excreted in urine (Wilson et al., 2019). In addition. As a member of cytochrome P450, CYP3A exhibits retinoic acid hydroxylase activity, which can promote the breakdown of retinoic acid (Martini and Murray, 1993). In our study, downregulation of *AOX1* and *CYP3A5* in capon liver predicts increased vitamin storage capacity. Reaction between the phenolic hydroxyl group on the 3-position of the pyridine ring of vitamin B6 and oxygen free radicals, thereby quenching reactive oxygen species and reducing oxidative damage (Wondrak and Jacobson, 2012). Vitamin A binds to its nuclear receptor, the retinoic acid X receptor (RXR), which regulates specific immune system cell subsets such as macrophages, dendritic cells, T cells, B cells and regulates phase function. Low doses of retinol can cause inflammation (Shojadoost et al., 2021). Therefore, increased storage capacity of vitamin A and B6 in liver of capon has a good effect on the immune system and antioxidant capacity.

### Differences in spleen transcriptomic profiles between capons and roosters

Among the three tissues, the spleen was the most affected by castration in terms of the number of differentially expressed genes. Among them, platelet activating factor receptor (*PTAFR*) is a candidate gene. Although It is not the most differentially expressed gene, it is function was studied more thoroughly.*PTAFR* was known to play a significant part in diverse cancers and diseases (Ninio et al., 2004; Hou et al., 2018). As a kind of G-protein coupled receptor, *PTAFR* has been reported to bind to the platelet-activating factor, then plays a role in a number of biological pathways including inflammatory diseases, cardiovascular homeostasis as well as cancer (Damiani and Ullrich, 2016). Down-regulated *PTAFR* caused by castration may lead to a change to platelet-activating factor, which in turn exerts roles in platelet aggregation, stimulation of neutrophils and macrophages, inflammation and allergic responses (Pałgan and Bartuzi, 2015; Liu et al., 2017). Among the top 10 upregulated genes, Hemopexin (*HPX*) is better known than others, the researches on the function of the other genes is rare. HPX is a gene which encodes a plasma glycoprotein (hemopexin) that binds heme with high affinity, and widely participate in the regulation of various physiological and pathological processes (Dong et al., 2013). The up-regulated *HPX* gene may improve the ability of recovery of injury or disease (Ashouri et al., 2021). The removal of free heme by *HPX* helps protect numerous cellular and regulatory processes, such as macrophages, endothelial cells, and hepatocytes, by reducing heme toxicity and lipoprotein oxidation (Smith and McCulloh, 2015). Furthermore, HPX works with haptoglobin (HP) to simultaneously counteract downstream pro-oxidant and pro-inflammatory damage mediated by free heme and its precursor hemoglobin (Hb). By scavenging oxides from the circulation using HPX and HP, reactive oxygen species (ROS) production and downstream oxidative and inflammatory damage can be reduced. Upregulated HPX genes may alter the expression or activation of proinflammatory, clinically deleterious cytokines and transcription factors (Ashouri et al., 2021), Alleviates heme-related toxicity. The increase of *HPX* in the capon’s spleen may create beneficial immune conditions.

In spleen, most DEGs were enriched in biological process in the GO analysis. In biological process category, the most significant GO term was the regulation of cell differentiation. Among the top fold change DEGs enriched in the regulation of cell differentiation, Cytotoxic T lymphocyte antigen 4 (*CTLA4*; *CD152*) is involved in suppressing immune responses through a contact-dependent pathway. Regulatory T cells (Tregs) express large amounts of *CTLA4* and are considered to be a central pathway by which Tregs inhibit the activity of antigen-presenting cells (Wing and Sakaguchi, 2010). Likewise, lymphocyte activation gene 3 (*LAG3*, *CD223*) in Tregs, a CD4 homolog bound to MHC class II, binds MHC class II on immature dendritic cells to generate an inhibitory signal, Inhibition of dendritic cell maturation through contact dependence (Shevach, 2009). The continuous high expression of *CTLA4* and *LAG3* in avian thymic CD4^+^CD25^+^ cells suggests that this mechanism of contact-dependent induction of immunosuppression is active in avian Tregs. In our experiments, both *LAG3* and *CTLA4* were up-regulated in the spleen of capon. The interaction between androgens and Tregs has not yet been uniformly concluded, although it has been reported in the literature that testosterone can suppress immune function by inducing the expansion of Tregs (Fijak et al., 2011), But in mouse castration experiments, mice showed expansion of Tregs after castration (Tang et al., 2012). In KEGG analysis, hematopoietic cell lineage was the most significant enrichment, which indicates that the self-renewal and differentiation of hematopoietic stem cells may be affected by androgen level after castration. In our study, the top fold change DEGs enriched in this pathway including *ANPEP*, *CD7*, *CD2*, *CD24*, *ITGA2B*, *ITGB3*. *ANPEP*, also known as *ANP* or *CD13*, is able to modulate the development and function of immune-related cells (Lu et al., 2020). *CD13* has been implicated in monocytes/macrophage activation and differentiation (Koch et al., 1991). An increase of *CD13* expression also has been found during the differentiation from monocytes to macrophages (Koch et al., 1991). Meanwhile, blocking *CD13* by monoclonal antibodies or by binding of human cytomegalovirus (HCMV) inhibits macrophage differentiation (Gredmark et al., 2004). Moreover, *CD13* is involved in the development of dendritic cells from *CD34* ^*+*^ hematopoietic progenitor cells (Rosenzwajg et al., 2000), and is associated with the function of lymphocytes (Syrjälä et al., 1994; Spits et al., 1995), neutrophils (Fiddler et al., 2016), mast cells (Zotz et al., 2016), endothelial cells (Rangel et al., 2007; Yang et al., 2007) and fibroblasts (Morgan et al., 2015). *CD24* promotes the development of B cell (Duperray et al., 1990). The down-regulated genes included 14 DEGs including *CD2*, *CD7*, *ITGA2B* and *ITGB3*. The interaction of *CD2* and *CD3* is thought to be critical for T cell differentiation and functional activation (Rubin et al., 1992). Studies suggest that CD7 may be involved in activation and/or adhesion of T and NK cells (Carrera et al., 1988; Shimizu et al., 1992), and increase T cell IL-2 production and IL-2 receptor expression (Jung et al., 1992). *ITGA2B*, *ITGB3* are expressed in platelets and play a role in their aggregation (Phillips et al., 1988), While *ITGA2B* and *ITGB3* gene mutations are associated with Glanzmann thrombocytopenia (Nurden and Pillois, 2018). The results suggest that the development and function of T cell, NK cell and platelets were compromised, the activation and differentiation of monocytes/macrophages and development of B cell was increased. According to the up-regulated *LAG3* and *CTLA4* and their functions, we may explain the suppression of T cell in spleen of capon.

### Differences in hypothalamus transcriptomic profiles between capons and roosters

In hypothalamus, there were 15 DEGs detected between capon and rooster, and most of the DEGs were immune-related, of which *BLB2* was a typical gene associated with disease resistance. In chicken, the major histocompatibility complex (*MHC*) gene is located on chromosome 16, including NOR, BLA, Linkage group Y and Linkage group B (Salomonsen et al., 2003). Among them, Linkage group B is composed of closely linked polymorphic regions: BF (class I), BL (class II) and BG (Miller et al., 2004). In particular, the *MHC BLB2* gene located in the BL (class II) region is the main expressed gene and has a wide range of polymorphisms. The gene plays an important role in the presentation of extracellular antigens and the initiation of immune responses (Jacob et al., 2000), and was increasingly paid attention on the genetic variation (Xu et al., 2007). The correlation between high polymorphism in exon of the chicken *MHC BLB2* and disease resistance traits of chickens has also been proved (Boonyanuwat et al., 2006). The higher genetic diversity of *MHC BLB2* gene exon 2 in Hebei domestic chicken might be involved with its more robust disease resistance (Guo et al., 2012). In the current study, this result showed that castration has a great impact on immunity through hypothalamus. Additionally, hypothalamus is part of the central nervous system, which involved in the regulation of stress response and energy homeostasis (Bains et al., 2015). Since no stress-related changes were found in GO and KEGG analyses of hypothalamic DEGs in both groups, we assumed that the castration surgery may affect the immune- and stress-related indicators in chick period, but not for adult chicken when slaughtered.

Unlike the other two tissues, the DEGs in hypothalamus were mainly concentrated in cell component in the GO analysis. In the top 3 significant GO term, MHC protein complex and MHC class II protein complex accounted for two of them, which were related to chicken immunity. *BLB2* significantly enriched in the both GO terms. MHC I molecules help protect the host against infection and cancer by binding and presenting peptides to cytotoxic T cells (Sadasivan et al., 1996). The genetic region of the MHC class II has evolved to encode proteins that are critical for the adaptive arm of the immune system (Ohta et al., 2000). A total of 15 DEGs in hypothalamus mapped to 7 KEGG pathways. N-glycan-biosynthesis-related pathways were the most significant enrichment pathways. IgY is the major serum immunoglobulin of birds, reptiles and amphibians. Compared with mammalian IgG and IgE, IgY is more glycosylated because it contains two potential N-glycosylation sites. In antibody therapy, the structural characteristics of N-glycans are very important, because the properties of these glycans can decisively affect the therapeutic properties of antibodies. The carbohydrate moiety attached to a therapeutic antibody affects its thermal stability and physicochemical properties, as well as other key characteristics such as receptor binding activity, circulating half-life, and immunogenicity (Gilgunn et al., 2016). Therefore, the increased synthesis of N-glycans in capons may imply that IgY function in capons is actively regulated.

## Conclusion

The shear force of the breast muscle of capons was significantly lower than that of males, indicating that the tenderness of capons was better. Through GO enrichment and KEGG enrichment analysis of DEGs, we focused on the enrichment pathways and genes associated with oxidative detoxification, lipid metabolism, vitamin metabolism and immune function. Among them, the DEGs in the liver may play an important role in fat depositionand oxidative detoxification ability. The enriched pathways in the spleen indicated that DEGs have a potential role in activation and differentiation of monocytes/macrophages, heme detoxification, anti-inflammatory response, and B lymphocyte maturation. The DEGs in the hypothalamus were mostly immune-related genes, the N-glycan biosynthesis related pathways suggests that there are differences in IgY function between capons and roosters. Accordingly, castration-induced testosterone deficiency causes changes in fat deposition, vitamin storage, heme detoxification, hemoglobin concentrations, and innate and acquired immune cell function. Our results of the effects of castration on transcriptomic profiles in liver, spleen and hypothalamus provide new insights for better understanding of the genetic and biological changes in roosters in response to castration.

## Data Availability

The original contributions presented in the study are included in the article/Supplementary Material, further inquiries can be directed to the corresponding authors.
