# Supplementary material for: The transcriptome pattern of liver, spleen and hypothalamus provides insights into genetic and biological changes in roosters in response to castration
Source: Front Genet. 2022 Nov 9;13:1030886. doi: 10.3389/fgene.2022.1030886 (PMC9682263; doi:10.3389/fgene.2022.1030886)
Supplement: Supplementary file 1 [file DataSheet1.docx]

Table S1 Primer sequences of candidate validation genes

| Gene name | Primer sequences （5’-3’） | Product size （bp） | Amplification  efficiency |
| --- | --- | --- | --- |
| *FABP1* | F-CACCATTGGGGAAGAGTGTGA  R-GTTCGGTCACGGATTTCAGC | 122 | 103.2 |
| *A2ML1* | F-AGAGAGGCTCTGGGAGGAAG | 135 | 99.8 |
|  | R-GCACTGTCCGGGTAAAGACA |  |  |
| *SULT* | F-GAAGAGCTTCGGAGCGTGAT  R-CACCACCTTTGCGAAAGAGC | 106 | 102.6 |
| *EFCC1* | F-CTGTTCCGGGCAGTGGAG | 202 | 98.7 |
|  | R-CCACAGCAGCTTCATAGCCA |  |  |
| *ERMN* | F-GCTGGGGAAGAAGAACGACA  R-TTAAGCGATTCCCCACCTCG | 149 | 100.1 |
| *POLD4* | F-CACGAGAGCTGCTGGAGATG | 279 | 106.3 |
|  | R-GCGCTCTCCGCTTTATTCAC |  |  |
| *HPX* | F-GCATCTATGCCTTCCGTGGT | 170 | 103.2 |
|  | R-GTAGATGGAGACCTGCGAGC |  |  |
| *PTAFR* | F-GCTACAGCCTCTTCACCGTT  R-ACAGTGGCATCGTAACCAGG | 177 | 97.5 |
| *RN7SL1* | F-CACCAGGTTGCCTAAGGAGG  R-GTCTCTCTGTGTTGCCCAGG | 132 | 96.6 |
| *FEV* | F-AAGGCTTGGCAGATCCATCC  R-GGGAATCACCGCTCTCCAAA | 119 | 97.7 |
| *ST8SIA6* | F- GCTTCTAGGAACGGGCTCTG | 107 | 100.9 |
|  | R- ACTGTGGCCTGTTTCCCAAA |  |  |
| *COL28A1* | F- GGACCCAAGGGTTCTCAAGG | 239 | 97.2 |
|  | R- GGTCCTACCTGGCCTTGTTC |  |  |
| *β-actin* | F-GAGAAATTGTGCGTGACATCA  R-CCTGAACCTCTCATTGCCA | 152 | 99.3 |

Table S2 Characteristics of the reads from tissue libraries obtained from 2 groups.

| Sample | Raw reads | Clean reads | Total mapped | Multiple mapped^1^ | Uniquely mapped^2^ |
| --- | --- | --- | --- | --- | --- |
| H_R3 | 49891244 | 49232916 | 39522078(80.28%) | 1367073(2.78%) | 38155005(77.5%) |
| H_R2 | 53474500 | 52836622 | 42258536(79.98%) | 1789320(3.39%) | 40469216(76.59%) |
| H_R1 | 50236448 | 49643656 | 40033896(80.64%) | 1371349(2.76%) | 38662547(77.88%) |
| H_C3 | 54019288 | 53289462 | 42017365(78.85%) | 2188517(4.11%) | 39828848(74.74%) |
| H_C2 | 55158304 | 54437754 | 43856293(80.56%) | 1870613(3.44%) | 41985680(77.13%) |
| H_C1 | 55708796 | 54972042 | 44726912(81.36%) | 1777703(3.23%) | 42949209(78.13%) |
| S_R3 | 55871154 | 55154502 | 47058656(85.32%) | 2161348(3.92%) | 44897308(81.4%) |
| S_R2 | 53735830 | 52972832 | 44230391(83.5%) | 2228360(4.21%) | 42002031(79.29%) |
| S_R1 | 50997668 | 50297796 | 42821276(85.14%) | 1579303(3.14%) | 41241973(82.0%) |
| S_C3 | 53046282 | 52281246 | 42471442(81.24%) | 2750421(5.26%) | 39721021(75.98%) |
| S_C2 | 47609350 | 46731544 | 37411041(80.06%) | 2539691(5.43%) | 34871350(74.62%) |
| S_C1 | 45051960 | 44195700 | 36422921(82.41%) | 4776357(10.81%) | 31646564(71.61%) |
| L_R3 | 48076290 | 47539708 | 40018568(84.18%) | 2026727(4.26%) | 37991841(79.92%) |
| L_R2 | 51263214 | 50694478 | 41981040(82.81%) | 2541577(5.01%) | 39439463(77.8%) |
| L_R1 | 54709206 | 54069830 | 44416764(82.15%) | 2250400(4.16%) | 42166364(77.99%) |
| L_C3 | 46403902 | 45838772 | 37564854(81.95%) | 2452859(5.35%) | 35111995(76.6%) |
| L_C2 | 52984030 | 52302646 | 43474874(83.12%) | 2649513(5.07%) | 40825361(78.06%) |
| L_C1 | 55412512 | 54756506 | 45854667(83.74%) | 3611123(6.59%) | 42243544(77.15%) |

^1^Multiple mapped = number of clean reads and the ratio that matched two or more positions in the genome.

^2^Uniquely mapped = number of clean reads and the ratio that matched only one position in the genome.

H_R: hypothalamus of rooster; H_C: hypothalamus of capon; S_R: spleen of rooster; S_C: spleen of capon; L_R: liver of rooster; L_C: liver of capon


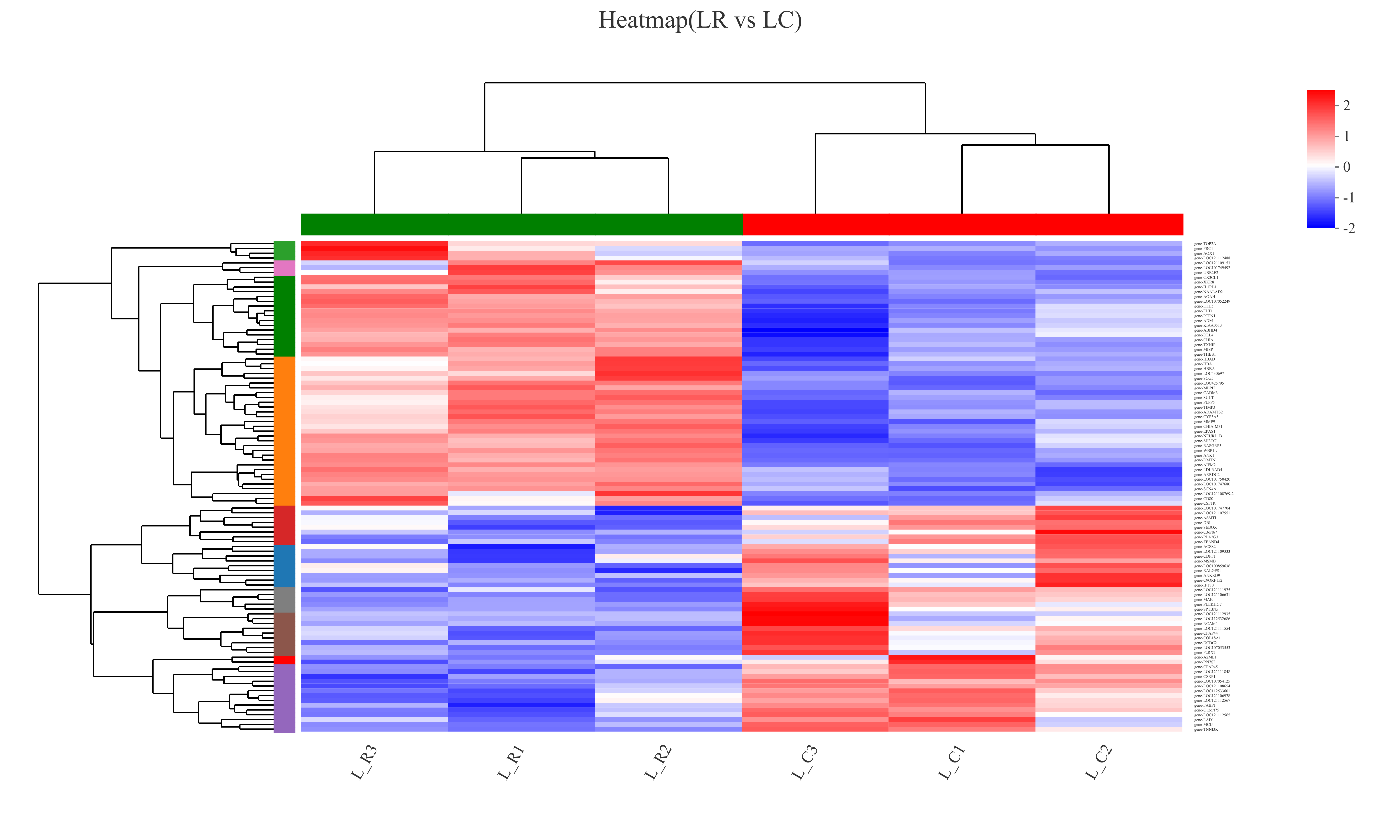


Figure S1. Cluster analysis of DEGs in livers between capon group and rooster group by the FPKM value. High expression genes are shown in red and low expression genes are shown in green. The closer the branches of the two samples are, the closer the expression patterns of all genes in the two samples are, and the closer the trend of gene expression are.

L_R: liver of rooster; L_C: liver of capon


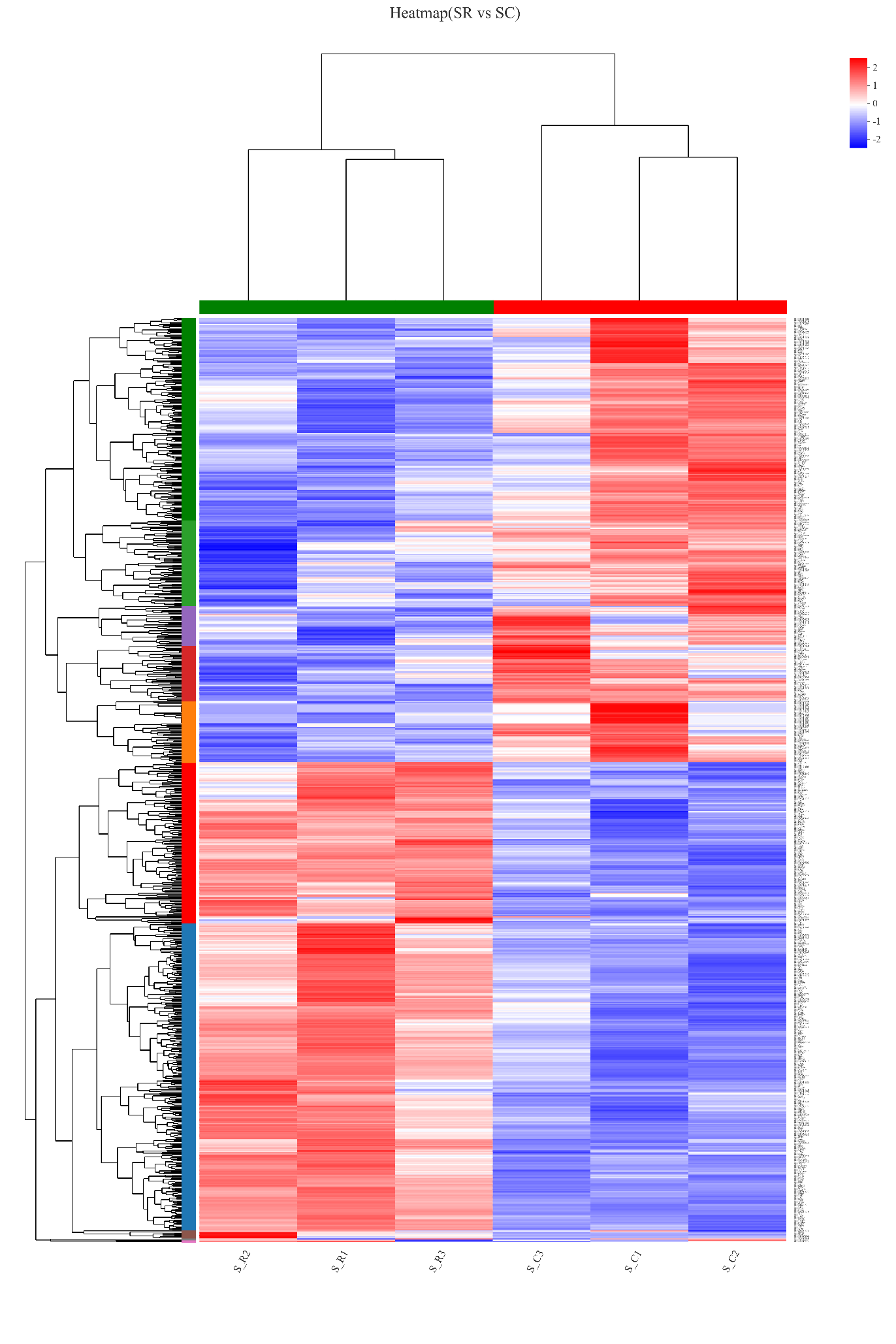
Figure S2. Cluster analysis of DEGs in spleens between capon group and rooster group by the FPKM value. High expression genes are shown in red and low expression genes are shown in green. The closer the branches of the two samples are, the closer the expression patterns of all genes in the two samples are, and the closer the trend of gene expression are.

S_R: spleen of rooster; S_C: spleen of capon


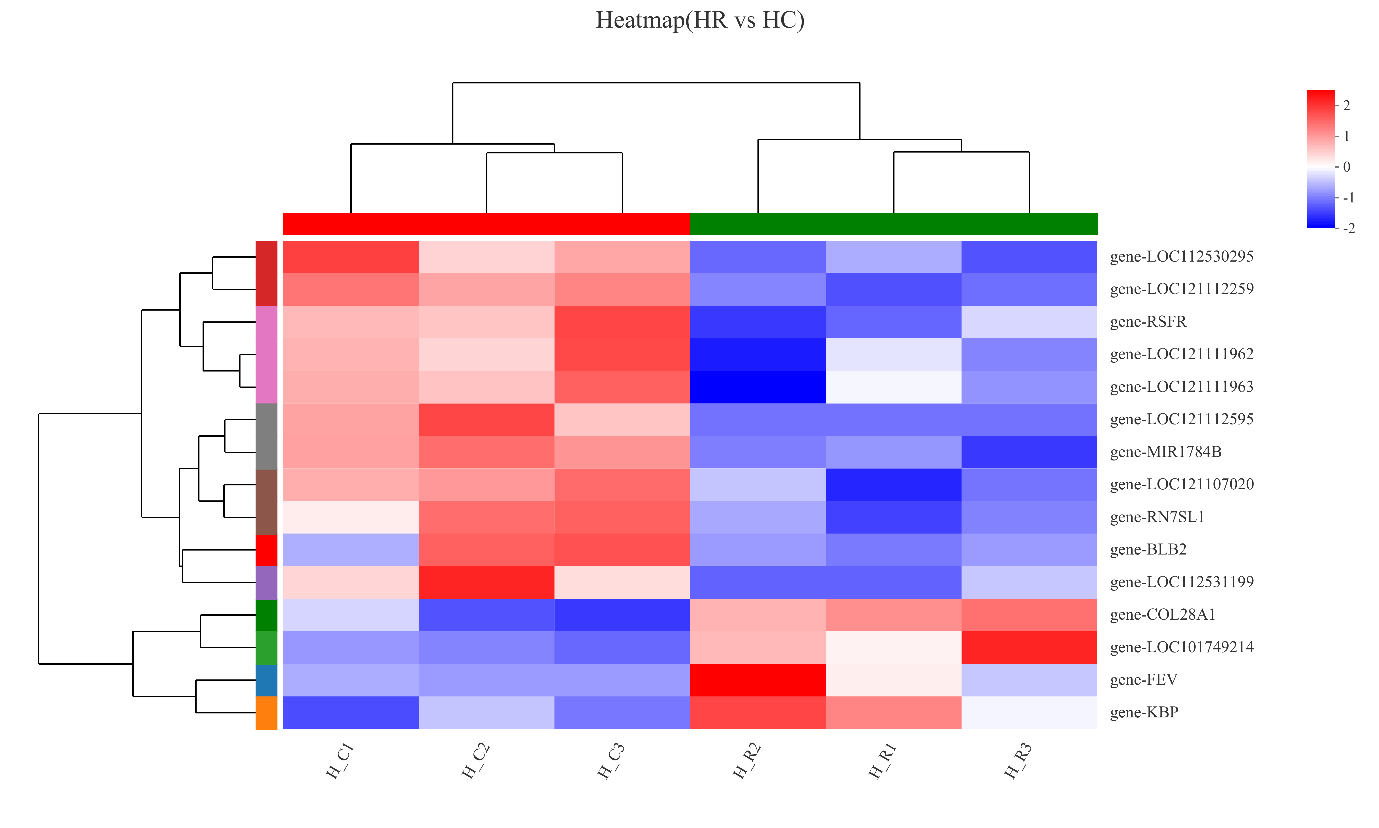
Figure S3. Cluster analysis of DEGs in hypothalamus between capon group and rooster group by the FPKM value. High expression genes are shown in red and low expression genes are shown in green. The closer the branches of the two samples are, the closer the expression patterns of all genes in the two samples are, and the closer the trend of gene expression are.

H_R: hypothalamus of rooster; H_C: hypothalamus of capon

The data presented in the study are deposited in the NCBI repository, accession number: SRP404882.
